# Myelinated axon physiology and regulation of neural circuit function

**DOI:** 10.1002/glia.23665

**Published:** 2019-06-24

**Authors:** Daumante Suminaite, David A. Lyons, Matthew R. Livesey

**Affiliations:** ^1^ Centre for Discovery Brain Sciences University of Edinburgh Edinburgh UK

**Keywords:** activity, axon sub‐domains, ion channels, myelin, myelinated axon plasticity

## Abstract

The study of structural and functional plasticity in the central nervous system (CNS) to date has focused primarily on that of neurons and synapses. However, more recent studies implicate glial cells as key regulators of neural circuit function. Among these, the myelinating glia of the CNS, oligodendrocytes, have been shown to be responsive to extrinsic signals including neuronal activity, and in turn, tune neurophysiological function. Due to the fact that myelin fundamentally alters the conduction properties of axons, much attention has focused on how dynamic regulation of myelination might represent a form of functional plasticity. Here, we highlight recent research that indicates that it is not only myelin, but essentially all the function‐regulating components of the myelinated axon that are responsive to neuronal activity. For example, the axon initial segment, nodes of Ranvier, heminodes, axonal termini, and the morphology of the axon itself all exhibit the potential to respond to neuronal activity, and in so doing might underpin specific functional outputs. We also highlight emerging evidence that the myelin sheath itself has a rich physiology capable of influencing axonal physiology. We suggest that to fully understand nervous system plasticity we need to consider the fact that myelinated axon is an integrated functional unit and adaptations that influence the entire functional unit are likely to underpin modifications to neural circuit function.

## 1 INTRODUCTION

Brain plasticity allows us to adapt, learn and refine our behaviors and skills, and is underpinned by modification to the structure and function of the nervous system. For decades the presumption was held that dynamic modification of neural circuits was solely due to modulation to the structure and function of neurons, with primary research focus being on synaptic plasticity. However, we now know that central nervous system (CNS) glia also serve as major regulators of neuronal network formation and function (see review Allen & Lyons, 2018). Although astrocytes regulate many aspects of neurophysiology, it is now clear that cells of the oligodendrocyte lineage are also responsive to neuronal activity. Given that oligodendrocytes are the myelinating cells of the CNS, such modifications are likely to affect neural circuit function, which has led to the model that dynamic regulation of myelination might represent a form of functional plasticity (see reviews Fields, 2015; Almeida & Lyons, 2017). The ability of myelin to facilitate the rapid saltatory conduction of action potentials (APs) along axons has been known for decades, but it is only now becoming clear that distinct axons can have specific patterns of myelin sheath number, distribution, length, and thickness along their length, and that these may be highly tuned to particular functional outputs (Almeida & Lyons, 2017). Therefore, the dynamic regulation of myelination may lie at the very heart of the nervous system plasticity, as per the topic of this special issue.

**Figure 1:**
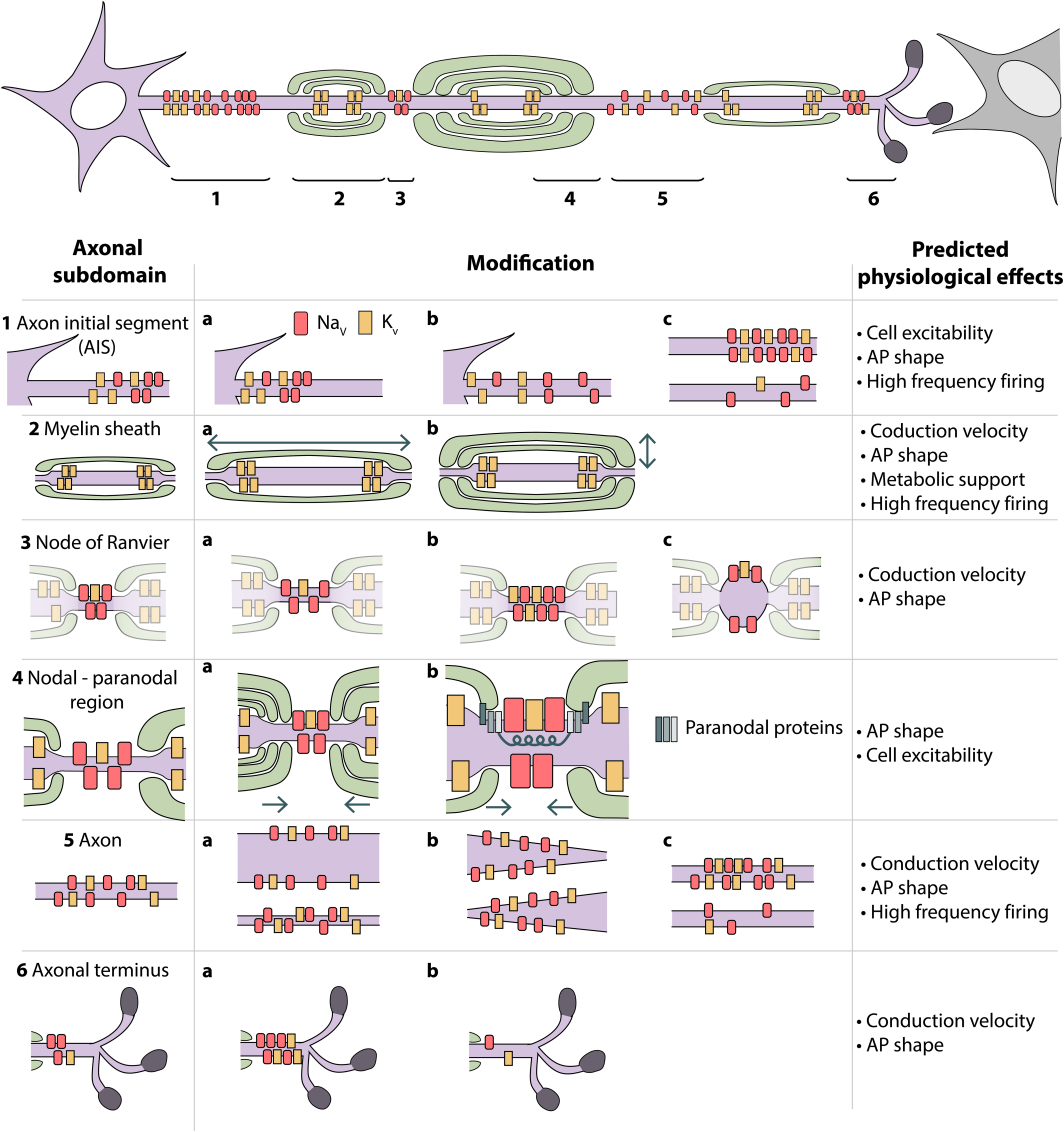
Overview of a neuron and its axonal subdomains, their possible modifications and predicted effects on axonal physiology (table). (1) Axon initial segment has been observed to change its position along the axon (a), alter its length (b), and ion channel density (c). (2) Myelin sheaths have been reported to change their length (a) and thickness, by increasing the number of wraps around the axon (b). (3) Nodes of Ranvier could increase their length (a), ion channel concentration (b) and diameter (c). (4) Within the nodal‐paranodal region the length and ion channel density of the node of Ranvier may change upon changes to myelin (a), or via an inside‐out mechanism, whereby the axonal cytoskeleton fine tunes the morphology and composition of the region (b). (5) The axon itself can vary in diameter (a, b) as well as alter ion channel density in unmyelinated segments. (6) In the axon terminus regions, the concentration of ion channels can be increased (a) or decreased (b) at the heminode

Many aspects of myelin plasticity/ adaptive myelination/myelinated axon plasticity have been considered extensively elsewhere, including how neuronal activity affects various aspects of oligodendrocyte lineage progression, from the proliferation of oligodendrocyte progenitor/precursor cells (OPCs), through to the differentiation and survival of oligodendrocytes, and their subsequent myelination (Almeida & Lyons, 2017; Káradóttir & Kuo, 2018; Monje, 2018). In this review, however, we aim to take a more axonal and physiological perspective, and we put forward the premise that to fully understand how regulation of myelination might affect neural circuit function we must consider the entire myelinated axon as the key functional unit, given that dynamic regulation of any one component of this unit is likely to affect others. The functional output of the myelinated axon unit reflects the organization and physiology of all its components; its axon initial segment, myelin sheaths, nodes of Ranvier, paranodes, juxtaparanodes, and even the morphology of the axon itself (Figure 1). Modulation to any or all of these components could, in principle, influence how APs arrive at presynaptic termini, which will in turn affect synaptic communication and circuit function. Here, we will review evidence that indicates that neuronal activity can regulate essentially all components of the myelinated axon unit, and we will consider how modulation of each component might influence conduction and axonal physiology. In the second part of our review, we will focus on recent studies that have started to reveal a rich repertoire of physiological interactions at the axon‐myelin interface, which are also likely to influence the function of the myelinated axon unit.

## 2 THE AXON INITIAL SEGMENT

In the axons of CNS neurons, the initiation of APs occurs at the axon initial segment (AIS), following the summation of integrated synaptic activity within the somatodendritic region (see review Kole & Stuart, 2012). The AIS represents an unmyelinated region between the axon hillock and the first myelin sheath in myelinated axons (Palay, Sotelo, Peters, & Orkand, 1968), where ion channel composition, identity, position, and density serve to regulate both AP initiation and subsequent conduction (Figure 1). The AIS typically contains a high density of voltage‐gated Na^+^ channels, e.g. Na_v_1.6 in mature neurons, and Na_v_1.2 in immature neurons; (Chomiak & Hu, 2009; Duflocq, Chareyre, Giovannini, Couraud, & Davenne, 2011; Duménieu, Oulé, Kreutz, & Lopez‐Rojas, 2017; Rush, Dib‐Hajj, & Waxman, 2005; Van Wart, Trimmer, Matthews, Trimmer, & Matthews, 2007), critical for axonal excitability and AP initiation (Hu et al., 2009; Lorincz & Nusser, 2008; Rush et al., 2005). Furthermore, the AIS contains a high density of voltage‐gated K^+^ channels, which regulate intrinsic firing, excitability (K_v_7.2 and K_v_7.3 channels—“M‐channel”; Shah, Migliore, Valencia, Cooper, & Brown, 2008), as well as AP firing and waveform (K_v_1.1 and K_v_1.2; Kole, Letzkus, & Stuart, 2007). Correspondingly, disruption of ion channel localization at the AIS can alter cell excitability and AP shape *in vivo* (Zonta et al., 2011).

Importantly from the perspective of functional plasticity, the AIS has been shown to be rapidly responsive to changes in neuronal activity, whereby changes can occur within a matter of hours (Evans, Dumitrescu, Kruijssen, Taylor, & Grubb, 2015). Increased neuronal activity in dissociated primary hippocampal neurons shifts the AIS further away from the neuronal cell body, with this change reversible upon reducing activity (Grubb & Burrone, 2010). This AIS modification alters the neuron's excitability and is thought to represent a form of homeostatic plasticity that could maintain gross firing patterns in neural circuits, and prevent runaway activity that could result from recurrent strengthening of synaptic connections. In contrast, stimulation of GABA‐ergic neurons causes AIS relocation toward the soma and significant elongation (Chand, Galliano, Chesters, & Grubb, 2015). In further evidence of activity‐driven regulation of the AIS, blocking neuronal activity in the avian auditory system results in AIS elongation and increased expression of Na_v_ channels, which serves to increase neuronal excitability (Kuba, Oichi, & Ohmori, 2010). Therefore, it is emerging that the AIS can be regulated by different forms of activity in distinct neuronal subtypes. However, while such adaptive changes are highly likely to affect neural circuit function, how activity‐driven AIS‐specific adaptations ultimately affect the functional output of the myelinated axon unit at presynaptic termini remains to be determined.

## 3 MYELIN

Myelin acts as an electrical insulator and decreases axonal capacitance, altering conduction properties and reducing the energy requirements for action potential propagation. As a result, large increases in conduction velocity are gained relative to unmyelinated axons of the same size when the fiber diameter is above 0.2 μm (Waxman & Bennett, 1972). Arguably, of principle functional importance is the fact that myelin sheath formation coincides with the redistribution of ion channels along the axon. In an unmyelinated or pre‐myelinated axon, Na_v_ channels are distributed uniformly along the axon (Caldwell, Schaller, Lasher, Peles, & Levinson, 2000; Van Wart et al., 2007; Westenbroek, Merrick, & Catterall, 1989), which permits only slow AP propagation. With the formation of myelin sheaths, Na_v_s along with many other ion channels become spatially restricted to the nodes of Ranvier between consecutive myelin sheaths and others at juxtaparanodes, which is the myelinated axon subdomain underneath the myelin sheath at the other side of the paranodal junction (Figure 1). Due to the organization of ion channels along the myelinated axon, the AP is regenerated only at the nodes of Ranvier, thereby greatly increasing AP conduction velocity in an energy efficient manner known as saltatory conduction (Harris & Attwell, 2012; Tasaki, 1940; Wang et al., 2008). How dynamic regulation of the structure and composition of nodes, paranodes and juxtaparanodes might affect conduction will be examined in more detail in coming sections. First we review our current, and surprisingly limited, understanding of how modulation of myelination can affect conduction.

The historical textbook view of the myelinated axon was that myelinated axons were fully myelinated along their length, that the length and thickness of myelin sheaths along axons was consistent, that sheath length is roughly equivalent to about 100X axon diameter (Hess & Young, 1949), and that larger diameter axons have thicker myelin sheaths (Donaldson & Hoke, 1905). Based on such presumptions, initial modeling studies predicted that myelin sheath length correlated positively with conduction velocity (Hursh, 1939; Moore, Joyner, Brill, Waxman, & Najar‐Joa, 1978; Rushton, 1951), but only to a flat maximum, beyond which further increases in length would not result in gains in speed (Huxley & Stampfli, 1949). This premise was validated recently through analyses of animals with short myelin sheaths in the peripheral nervous system (PNS) (Wu, Williams, Delaney, Sherman, & Brophy, 2012), but our understanding of how sheath length and conduction are related in the CNS remains less clear.

Although early modeling studies also held that the fastest conduction speed represented the optimal state, it is now emerging that the precise timing of conduction rather than maximizing speed might be optimal with respect to function, particularly in the CNS (Pajevic, Basser, & Fields, 2014; Seidl, 2014). For example, in many circuits, conduction times appear highly regulated, for example, to allow the synchronous arrival of APs at specific targets (Salami, Itami, Tsumoto, & Kimura, 2003), with myelination playing a role in regulating the temporal precision of AP delivery (Lang & Rosenbluth, 2003). It is now also clear that myelinated axons in the CNS can have very variable patterns of myelination along their length (Auer, Vagionitis, & Czopka, 2018; Hill, Li, & Grutzendler, 2018; Micheva et al., 2016; Stedehouder et al., 2017; Tomassy et al., 2014). For example, CNS myelin sheaths are often much shorter than in the PNS, and modeling has suggested that variation in the length of such short sheaths might have significant effects on conduction velocity (Brill, Waxman, Moore, & Joyner, 1977; Ford et al., 2015; Moore et al., 1978). In fact, recent anatomically informed electrophysiology and modeling has suggested that even modest elongation of short myelin sheaths may actually begin to reduce conduction speed (Ford et al., 2015). In the auditory system, where precise timing is essential for circuit function (Seidl, Rubel, & Barria, 2014), myelin sheath length appears highly tuned to mediate precise conduction times (Ford et al., 2015; Seidl et al., 2014), including, quite remarkably, along distinct axonal branches of a single neuron (Seidl & Rubel, 2016). Such observations indicate that local adaptive mechanisms might influence sheath length and in turn conduction. Supporting such a premise, is emerging evidence that neuronal activity can indeed directly regulate the length of myelin sheaths both *in vitro* and *in vivo* in zebrafish and rodents (Fields et al., 2015; Hines, Ravanelli, Schwindt, Scott, & Appel, 2015; Koudelka et al., 2016). Furthermore, recent live‐imaging studies in zebrafish indicate that distinct localized codes of Ca^2+^ activity in myelin sheaths prefigure sheath elongation and shrinkage, and that at least some of these myelin Ca^2+^ signatures are driven by neuronal activity (Baraban, Koudelka, & Lyons, 2018; Krasnow, Ford, Valdivia, Wilson, & Attwell, 2018). Therefore, distinct forms of neural activity might fine‐tune sheath growth along axons, which may have the consequence of influencing conduction velocity. However, only one study, to our knowledge, has investigated how regulation of sheath length following manipulation of neuronal activity affects conduction velocity (Etxeberria et al., 2016), but this followed a manipulation that affected additional myelin parameters, highlighting the need to experimentally disentangle the mechanisms of activity‐regulated myelination, to enable specific investigation of the effects on function.

The multi‐lamellar and lipid rich nature of myelin sheaths increases insulation by preventing current leakage from the axon (Bakiri, Káradóttir, Cossell, & Attwell, 2011), and decreases capacitance, allowing more rapid charging of nodes. Together, these properties help explain the observation that thicker myelin sheaths are generally associated with more rapid conduction in both experimental scenarios (Sanders & Whitteridge, 1946; Verhoeven et al., 2003) and computer simulations (Berthold, Nilsson, & Rydmark, 1983; Goldman & Albus, 1968; Moore et al., 1978; Rushton, 1951; Smith & Koles, 1970). Initial modeling predicted that the most rapid conduction would be observed when a ratiometric measure of myelin thickness called the g‐ratio (the ratio of the axon diameter and the myelin + axon diameter) was 0.6 (Goldman & Albus, 1968; Rushton, 1951). However, in reality, myelin thickness along axons can be highly heterogeneous, with most measurements of myelin thickness in the CNS deviating significantly from this g‐ratio value of 0.6; typically being higher, that is, having thinner sheaths (Chomiak & Hu, 2009). Although the relationship between regulation of myelin thickness and the function of specific axons and circuits remains to be assessed in detail, evidence is emerging that myelin thickness is also adaptable and can be regulated by experimentally and physiologically‐driven changes in neuronal activity (Gibson et al., 2014; Liu et al., 2012; Makinodan, Rosen, Ito, & Corfas, 2012; Mitew et al., 2018). In addition to modulation of myelin sheath length and thickness, neuronal activity has also been shown capable of influencing the formation of myelin sheaths on axons, by biasing myelination toward more active axons. However, activity is not required for the myelination of any axons examined to date per se, and it remains unclear to what extent the activity‐regulated aspects of myelin formation affect conduction.

Indeed, many questions remain to be addressed regarding activity‐regulated myelination: do changes in myelin sheath number, distribution, length, and thickness all reflect localized interactions between active axons and myelin sheaths? Do similar mechanisms underpin distinct aspects of activity‐regulated myelination? Can activity‐driven changes to myelination occur only during the initial myelination of an axon, irrespective of whether this occurs during development or in adulthood, or can activity regulate the myelination of mature myelinated axons or myelin sheaths? Most importantly, how do any forms of activity‐regulated myelination affect circuit function? Prior to addressing this last question, we will need to understand the molecular mechanisms by which neuronal activity regulates myelin sheath formation, growth and remodeling along axons *in vivo*.

## 4 NODES OF RANVIER

In addition to patterns of myelination that appear to correlate with specific conduction properties, there is now evidence that the fine parameters of nodes of Ranvier may also be tuned to mediate specific functions. For example, Globular Bushy Cell axons in the auditory system have distinctly different node diameters along their length, increasing systematically towards the axonal terminus (Ford et al., 2015), with a larger nodal diameter allowing a greater surface area for Na_v_s to localize and facilitate increased sodium currents. Such alterations in nodes have also been predicted to affect conduction and mediate the timely innervation of the giant calyx of Held synapse in this auditory circuit. Together with observations that node sizes vary more between than along axons in both the optic nerve and the corpus callosum (Arancibia‐Cárcamo et al., 2017), evidence is emerging that axon‐specific differences in nodal geometry may be widespread. Modeling based on these observations of node size distribution predict that changing nodal geometry are likely to affect conduction along single axons. These calculations proposed that if the total number of ion channels in nodes stays constant, increasing nodal length along axons should slow AP conduction velocity, by increasing the nodal charging time whereas shortening node length was predicted to speed up conduction velocity. In contrast, if ion channel density stayed constant upon nodal length changes, the opposite outcomes were predicted, with increasing length speeding up conduction and decreasing it slowing it down (Arancibia‐Cárcamo et al., 2017). At present, it remains unclear whether nodal geometry is dynamically adaptive* in vivo*, but this seems plausible given that changes to nodal organization may result from subtle regulation of myelin sheath elongation, retraction, or wrapping (but see Paranode and Juxtaparanode section below).

Detailed knowledge on the ion channel composition at the node of Ranvier and how core molecular mechanisms by which nodes are formed has been reviewed elsewhere, for example, Susuki & Rasband, 2008, Rasband & Peles, 2016. With respect to modulation of conduction velocity it is important to note that dynamic regulation of nodal ion channel composition is also likely to affect function, in addition to proposed effects of nodal geometry. For example, Na_v_1.6 type channels, which are highly enriched in nodes of the healthy and mature CNS (Boiko et al., 2001; Caldwell et al., 2000), are thought to have properties well suited to AP regeneration along the axon, and their nodal density correlates with significant increase of conduction velocity (Rasband et al., 1999). However, the repertoire of Na_v_s localized to nodes has been shown to vary depending on neuronal cell subtype (Arroyo et al., 2002; Duflocq, Le Bras, Bullier, Couraud, & Davenne, 2008), maturation, myelination (Boiko et al., 2001; Duflocq et al., 2008), and region‐specific location within the nervous system (Duflocq et al., 2008). Similar regulation of other ion channels that localize to nodes may also occur. For example, different types of potassium channel that play a role in AP repolarization and neuronal excitability (Devaux, 2004; Devaux et al., 2003; Pan, 2006; Schwarz et al., 2006), may be adapted to mediate specific conduction properties. Furthermore, although not yet extensively studied to the same level as the AIS, there is indeed evidence that neuronal activity‐dependent mechanisms at the node of Ranvier can lead to the regulation of the expression and function of ion channels that affect AP conduction (Gründemann & Clark, 2015). What remains quite unclear is how different patterns of neuronal activity regulate the distinct components of the myelinated axon, and how responsive to activity different components are at specific stages of the formation and function of the unit.

## 5 PARANODES AND JUXTAPARANODES

Paranodes flank both sides of the node of Ranvier and contain septate‐like junctions that adhere the myelin sheath to the axon (Figure 1) via a cell adhesion complex formed by glial‐Neurofascin binding to axonally‐expressed Contactin‐1 and Contactin‐associated protein 1 (Caspr1). Paranodes are essential for nodal stability (Amor et al., 2017; Desmazieres et al., 2014; Taylor, Saifetiarova, & Bhat, 2017; Zonta et al., 2008) and act as physical barriers that prevent the diffusion of nodal proteins including ion channels away from the node (Pillai et al., 2009). As noted above, very small changes in myelin sheath elongation or retraction could elicit relatively large changes with respect to node size by altering paranode position or dynamic activity. However, recent evidence indicates that subtle changes in the geometry of the region spanning the node and its adjacent paranodes might actually be regulated by the axonal cytoskeleton (Figure 1). This inside out (axon to myelin) model proposes that axonal spectrins bridge the paranodal‐nodal‐paranodal (PNP) domain and that protein 4.1B links these spectrins to the C‐terminal of Caspr at the paranodal domain (reviewed in Ghosh, Sherman, & Brophy, 2018). The extracellular domain of Caspr in turn provides the anchor to the myelin sheath through its interaction with glial neurofascin (Figure 1). Therefore, if the axonal cytoskeleton constricts within the PNP domain, this could bring the myelin sheaths closer together via the axon‐mediated anchorage at the paranodes or, conversely, allow them to slip further apart. Indeed, when either the C‐terminus of Caspr or protein 4.1B is missing from the axon, node of Ranvier formation is significantly delayed, during which time longer nodes are observed (Brivio, Faivre‐Sarrailh, Peles, Sherman, & Brophy, 2017). These data represent another example of how inextricably associated the regulation of myelin and axon biology is. It remains unknown if the axon cytoskeleton at the PNP region can be regulated by neuronal activity. However, it is of note that subtle dysregulation of paranodes and elongation of nodes is a hallmark of myelinated axon pathologies and ageing (Calvo et al., 2016): therefore, dysregulation of the PNP domain may contribute to functional deficits.

Immediately adjacent to the paranodal region, on the myelin sheath side is the juxtaparanode (Bhat et al., 2001; Boyle et al., 2001), where axonal Shaker‐type voltage‐gated potassium channels K_v_1.1 and K_v_1.2 become localized upon myelin compaction (Baba et al., 1999; Rasband, Trimmer, Peles, Levinson, & Shrager, 2000; Wang, Kunkel, Martin, Schwartzkroin, & Tempel, 1993). The juxtaparanodal localization of axonal K_v_ channels has been suggested to regulate axonal excitability (Smart et al., 1998), AP regeneration (Kocsis, Waxman, Hildebrand, & Ruiz, 1982; Vabnick et al., 1999), and AP repolarization during both development (Vabnick et al., 1999) and following remyelination (M N Rasband et al., 1998). However, it has not yet been possible to specifically dysregulate the localization of K_v_ channels at the juxtaparanode, leaving their precise role(s) inferred. Furthermore, whether physiologically relevant activity‐dependent changes in ion channel composition or expression occur at the juxtaparanode remains unknown, although this can occur following induction of pathological states of activity (Calvo et al., 2016). In the “Myelin physiology and regulation of conduction and function” section below, we focus on how the myelin sheath may play a critical role in buffering the K^+^ ions released by juxtaparanodal K_v_ channels, highlighting yet again the integrated nature of the myelinated axon unit and the interconnectedness of its functional domains.

## 6 AXON CALIBER

In addition to myelin and the axon sub‐domains described above, another major determinant of conduction velocity along myelinated axons is the morphology of the axon itself, and in particular its cross‐sectional size, or caliber. Axons in the CNS vary in cross‐sectional size (caliber) by over 100‐fold in diameter or 10,000‐fold in area (Perge, Niven, Mugnaini, Balasubramanian, & Sterling, 2012). Although not an absolute relationship *in vivo*, in general CNS axons under 0.5 μm in diameter are unmyelinated, and those over that value myelinated, with some myelinated axons in the vertebrate CNS exceeding 50 μm in diameter (Klingseisen & Lyons, 2017; Perge et al., 2012). Axon caliber is in and of itself a signal that can regulate both myelin sheath formation and growth (Bechler, Byrne, & Ffrench‐Constant, 2015; Lee et al., 2012; Mayoral, Etxeberria, Shen, & Chan, 2018), and in the PNS at least, myelin in turn reciprocates and supports the continued growth of axons in caliber (Sherman et al., 2012). Once again such bidirectional interactions indicate that adaptive changes to either axons or myelin are likely interdependent.

In myelinated axons conduction velocity increases proportionally with increasing axon diameter (Waxman & Bennett, 1972), because larger diameter leads to a reduced axial resistance and an increase in the inward ionic current, due to larger surface area. In addition to regulating conduction velocity, axons of different caliber have also been proposed to have distinct capacities to sustain high‐frequency firing of APs (Perge et al., 2012) (Figure 1 **)**, although this remains to be experimentally validated. Axons can also exhibit notable changes in caliber along their length (Greenberg, Leitao, Trogadis, & Stevens, 1990) and such variation has also been predicted to have the capacity to change conduction velocity along axons (Goldstein & Rall, 1974). In the avian auditory system, differences in axonal diameter between axon branches of the same neuron, but of different length, has been associated with tuning of conduction velocities (in conjunction with specific patterns of myelination) to allow simultaneous AP arrival at target neurons (Seidl & Rubel, 2016). In addition, it is well‐known that there are conspicuous constrictions in axon calibre at the PNP region, which have also been predicted to regulate conduction velocity (Halter & Clark, 1993).

Recent studies have indicated that like many other aspects of the myelinated axon, axon diameter is also adaptable and can change in response to neuronal activity. In one super‐resolution‐based study of cultured unmyelinated axons, it was found that both synaptic boutons and axons rapidly increase in diameter following high‐frequency neuronal stimulation (Chéreau, Saraceno, Angibaud, Cattaert, & Nägerl, 2017). Interestingly, the observed increase in the synaptic bouton size was temporary and predicted to decrease AP conduction velocity, whereas the longer‐term increase in axon caliber was predicted to speed up AP propagation. *In vivo* data supporting the premise that neuronal activity can regulate caliber along myelinated axons has recently come from findings that axon diameter increases with the onset of hearing and that blocking auditory input resulted in axons growing to smaller diameters (Sinclair et al., 2017). However, changes to myelination were also observed with both hearing onset and sensory deprivation, highlighting yet again the need to experimentally disentangle how specific forms of activity might coordinately or differentially affect axon caliber, myelination, and axon domains along myelinated axons and in intact circuits.

## 7 HEMINODES AND AXON TERMINI

At the end of the terminal myelin sheath, prior to axon termini, Na_v_ channels are densely clustered in so‐called heminodes (Figure 1). Transient, persistent and resurgent Na_v_‐mediated currents, originating from the axon heminode, are critical for the reliability and temporal fidelity of presynaptic spikes (Huang & Trussell, 2008; J. Hee Kim, Kushmerick, & von Gersdorff, 2010). Animal models with no compact myelin exhibit a reduced reliability of presynaptic firing and impaired temporal fidelity of synaptic transmission during high‐frequency firing, thought to be primarily because of dysregulation of Na_v_‐channel localization at the terminal heminode (Berret, Kim, Lee, Kushmerick, & Kim, 2016; J. H. Kim, Renden, & von Gersdorff, 2013). Importantly, heminodal ion channel composition is activity‐dependent, indicating that heminodes might be accutely tuned to mediate specific conduction properties (Grubb & Burrone, 2010; Xu, Berret, & Kim, 2017). In addition to ion channel regulation, the heminode also appears to be geometrically regulated. For example, distinct types of Globular Bushy Cell axon within the auditory brainstem have distinct heminode diameter innervating the giant calyx of Held (Ford et al., 2015). Despite being largely analogous to nodes of Ranvier in composition (Brivio et al., 2017), the physiological function of the heminode may be more directly coupled to the presynaptic function. Therefore, it is possible that heminodal regulation represents a major site of refinement of conduction properties and localized presynaptic function. Although many of the studies noted here have focused on heminodal regulation of the giant calyx of Held synapse, it is possible that heminodal regulation may be commonplace, and serve to regulate how APs along the myelinated axon, or branches thereof, are delivered to presynaptic termini. In addition, heminodes are present at proximal axonal regions of sensory neurons, where they shape circuit function (Wan & Corfas, 2017) and may also be present along myelinated axons with long unmyelinated stretches (Tomassy et al., 2014). However, the presence or roles of heminodes along partially myelinated axons remains to be fully investigated, as does investigation of any associated function.

The arrival of APs at the axon termini is critical to circuit function, as it is the principle driver of activity‐dependent vesicular transmitter exocytosis at the synapse (reviewed in Südhof, 2012). As is becoming increasingly apparent, the functional output of the myelinated axon unit is likely to deliver APs to the presynaptic terminal with appropriate temporal precision and at the necessary frequency according to meet specific requirements of distinct circuits. Recent observations made in Long Evans Shaker rats, which exhibit impaired myelination (due to disruption of myelin basic protein), have shown that myelination of cerebellar Purkinje axons critically regulates presynaptic terminal composition (fewer active zones) and thus function (Barron, Saifetiarova, Bhat, & Kim, 2018). Indeed, the Ca^2+^‐dependence of the kinetics of activity‐dependent presynaptic processes are tightly coupled to AP dynamics (Südhof, 2012) and therefore the output of myelinated axon units are intimately linked to vesicular release. Changes to AP waveform and the fidelity of AP firing via regulation of ion channel expression at the presynaptic terminal (Bean, 2007) or even long‐range effects of axonal excitability (Shu, Hasenstaub, Duque, Yu, & McCormick, 2006) can influence the size of calcium currents in the presynaptic terminal (Bischofberger, Geiger, & Jonas, 2006; Geiger, Jonas, & Der Universita, 2000), and in turn neurotransmitter release at the synapse (Sabatini & Regehr, 1997). Furthermore, the ability of presynaptic terminals to undergo activity‐dependent adaptation in response to AP activity is well‐established and appears key in specifically tuning the presynaptic vesicular release to the synapse (Castillo, 2012; Costa, Mizusaki, Sjöström, & van Rossum, 2017; Regehr, 2012). Also, the tuning of synaptic strength through long‐term plasticity (potentiation or depression) is highly dependent upon the temporal interval and the order of spiking activity of both presynaptic and postsynaptic synapses (spike‐time dependent plasticity, STDP) and is central to models of circuit‐level plasticity, development, and learning (Feldman, 2012). Myelinated axon output may be ideally suited to generate STDP in some circuits, and recent computational modeling indicates that physiological axonal spike propagation delays have the potential to yield novel neuronal activity and synaptic connectivity patterns, which cannot be captured by classic STDP models (Asl, Valizadeh, & Tass, 2018). Despite the fact that the nature of AP arrival (regulated entirely by the myelinated axon) influence presynaptic function, the role that regulation of any component of the myelinated axon, or the output of the entire unit plays in synapse function has been hugely understudied and represents a major challenge for the field.

## 8 MYELIN PHYSIOLOGY AND REGULATION OF CONDUCTION AND FUNCTION

**Figure 2:**
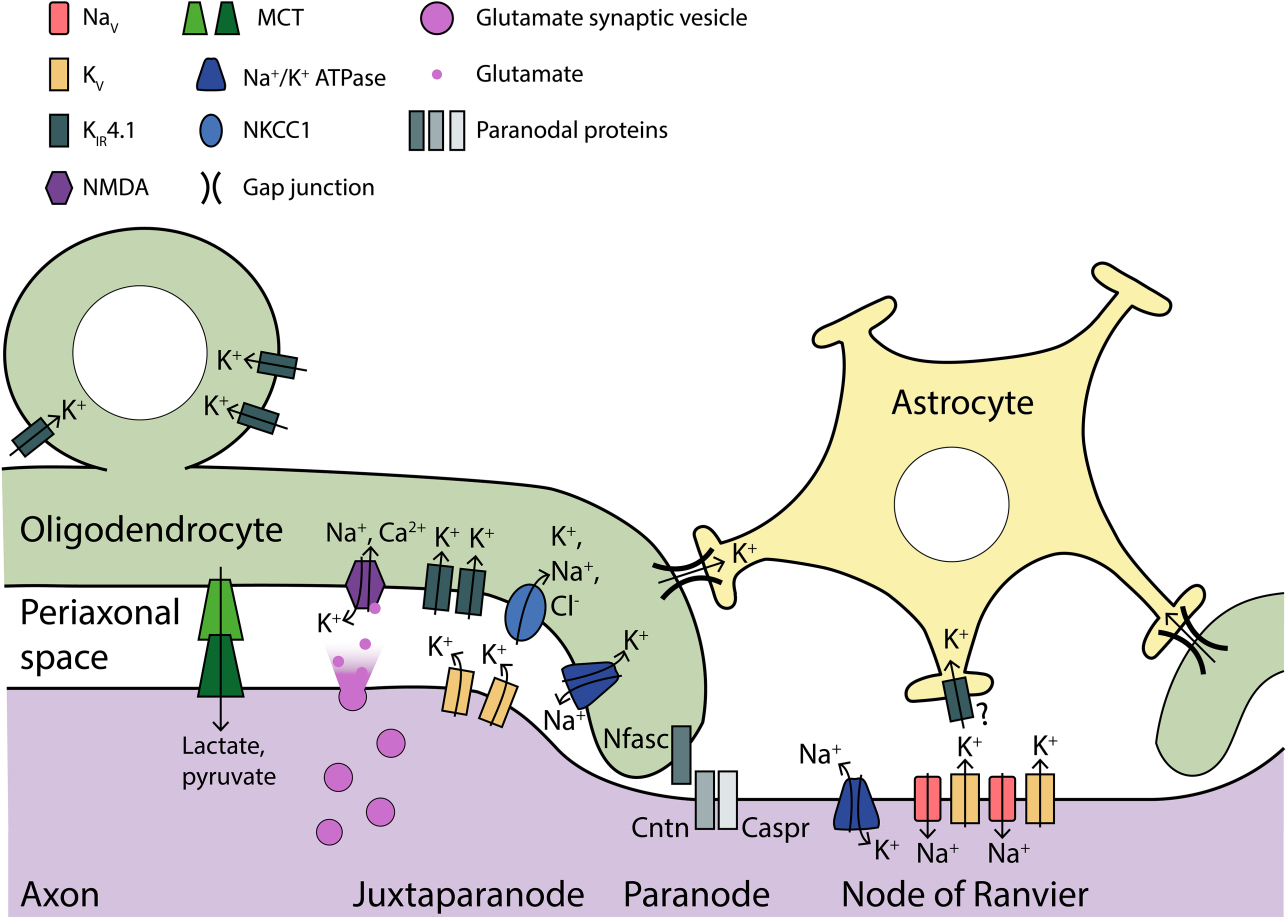
Ion channel/transporter expression contributing to axonal homeostasis. Following an action potential axonal K^+^ ions enter the periaxonal space via voltage‐gated potassium channels (Kv). In order to prevent elevated axonal excitability and prolonged excitotoxicity K^+^ is expected to be rapidly cleared from underneath the myelin sheath by myelinating oligodendrocytes via K_IR_, and possibly also Na^+^─K^+^─Cl^−^ cotransporter‐1 and/or the Na^+^/K^+^ ATPase. Excess of K^+^ in the oligodendrocyte is then passed on to astrocytes via gap junctions. At the nodes of Ranvier, K^+^ efflux is predicted to be buffered by axonal Na^+^/K^+^ ATPases and astrocytic K_IR_ channels. Paranodal proteins Neurofascin, contactin and Contactin‐associated protein (Caspr) separate nodal and juxtaparanodal regions and form a barrier preventing protein diffusion. Oligodendrocytes also provide metabolic support to axons via monocarboxylate transporters (MCTs) at the interface of the axon and myelin sheath

Studies are now emerging that indicate the direct interface between the myelin sheath and the axon is a site of potentially rich physiological interactions that may also influence myelinated axon function. This is evidenced partly by the fact that myelin sheaths have neurotransmitter receptors (Káradóttir & Attwell, 2007), ion channels and transporters (Annunziato, Boscia, & Pignataro, 2013; Philips & Rothstein, 2017) and exhibit localised codes of Ca^2+^ activity during sheath formation, growth, and remodeling (Baraban et al., 2018), at least some of which are under the control of neuronal activity (Krasnow et al., 2018). Recent work has also indicated that activity regulated responses of the oligodendrocyte may in turn affect axonal physiology by regulating metabolic support. Indeed, a host of studies have now led to a model that myelinating oligodendrocytes have the capacity to deliver metabolic substrates to axons via transporters localized at the interface of myelin sheaths and the axon (Figure 2), and that the metabolic support role may even represent the ancestral role of axonal ensheathment by glia (Fünfschilling et al., 2012; Lee et al., 2012). A recent study has proposed that this metabolic support may even be under dynamic regulatory control by neuronal activity (Saab et al., 2016), whereby NMDA receptors, known to be localized to the myelinating processes of oligodendrocytes (Káradóttir & Attwell, 2007), can respond to axonal glutamatergic release and trigger the redistribution of glucose transporters within the myelinating cell to regulate the metabolic support pathways mediated by the myelinating cell to the axon (Saab et al., 2016). This presents an elegant “supply–demand” mechanism by which oligodendrocytes sense axonal activity and then supply highly active axons, which therefore have increased energetic demand, with metabolic substrates. Although it remains to be determined how extensively employed this mechanism is during circuit function *in vivo*, this elegant premise highlights just how unexpectedly rich the potential roles of the myelinating glial cell are likely to be.

The topic of myelinating oligodendrocyte support of axons has been reviewed extensively elsewhere (Philips & Rothstein, 2017; Saab, Tzvetanova, & Nave, 2013), and so here we will focus on more recent evidence of how the physiology of the myelin sheath can directly affect axon conduction. The physiology of myelin sheaths and their impact upon the function of the myelinated axon unit are potentially extensive, but remain heavily underexplored. Therefore, this section will focus on the physiology of K^+^ clearance by myelinating oligodendrocytes given that this represents one of the better studied aspects of myelin physiology. As noted above voltage‐gated K^+^ channels are localized to the axonal juxtaparanode and are thought to mediate the vast majority of AP driven K^+^ efflux into the confined periaxonal space (Wang et al., 1993; reviewed in Salzer, 2003; Rash, 2010) (Figure 2). Indeed, *in silico* assessments predict that elevation of periaxonal K^+^ driven by high frequency neuronal activity could lead to various excitability states of the axon (Bellinger, Miyazawa, & Steinmetz, 2008; Bostock, Baker, & Reid, 1991; Brazhe, Maksimov, Mosekilde, & Sosnovtseva, 2011). Moreover, local elevations of K^+^ are likely to increase local excitability and the likelihood of excitotoxicity and, furthermore, osmotic swelling. Importantly, the myelin contains ion channels and transporters with the capacity, in principle, to dynamically regulate the composition of the periaxonal fluid. One of these myelin constituents is the inwardly rectifying K^+^ channel, *K*_*IR*_4.1, which has been shown to be enriched within the inner tongue of the myelin sheaths (Kalsi, Greenwood, Wilkin, & Butt, 2004; Poopalasundaram et al., 2000; Schirmer et al., 2018), thus ideally situated to respond to elevated K^+^ within the periaxonal space (Figure 2). In fact, *K*_*IR*_4.1 has been long hypothesized to be involved in periaxonal K^+^ clearance (Chever, Djukic, McCarthy, & Amzica, 2010; Djukic, Casper, Philpot, Chin, & McCarthy, 2007; Neusch, Rozengurt, Jacobs, Lester, & Kofuji, 2001), but only recently has its role been confirmed using a conditional *K*_*IR*_4.1 subunit knockout where its expression is specifically targeted in mature MOG‐expressing oligodendrocytes (Larson et al., 2018). Animals lacking *K*_*IR*_4.1 in mature oligodendrocytes showed a delayed recovery of compound AP amplitudes and an impaired return of the oligodendrocyte resting membrane potential to steady‐state in response to high frequency stimulation, consistent with an accumulation of extracellular K^+^. Furthermore, consistent with an increased neuronal excitability, mice exhibited behavioral deficits and increased mortality, likely associated with spontaneous seizures, showing that dysregulation of K^+^ clearance can give rise to pathological states. In a separate study, conditional knockout of *K*_*IR*_4.1, using a different cre driver (Cnpase) showed a more severe cellular pathology, including dystrophic optic nerve axons accompanied by delayed visual evoked potentials and impaired visual function (Schirmer et al., 2018). These differences may lie in the different brain areas investigated or the different transgenic strategies used to inactivate *K*_*IR*_4.1 function. Nonetheless, the evidence that *K*_*IR*_4.1 channels in myelin influence periaxonal K^+^ demonstrates that regulation of intracellular and extracellular concentrations of ions via ion channels and transporters is a major function of the mature oligodendrocyte plasma membrane. Given the key role of myelinating oligodendrocyte *K*_*IR*_4.1 channels in K^+^ buffering in the periaxonal space, it is clear that biophysical regulation and/or expression of oligodendrocyte *K*_*IR*_ *4*.1 channels have the potential to regulate axonal excitability. In line with a possible regulatory role for *K*_*IR*_4.1 channels in driving region/circuit‐specific conduction properties, spatial, and temporal heterogeneity in the expression of these channels in mature oligodendrocytes has been indicated (Poopalasundaram et al., 2000). In addition, *K*_*IR*_ channels also have the potential to be regulated by a range of cytoplasmic signaling molecules, including phosphatidylinositol 4,5‐bisphosphate (PIP_2_, reviewed in Hibino et al., 2010), the levels of which can influence myelin sheath growth, even in adulthood (Snaidero et al., 2014).

The importance of oligodendrocytes and myelin in such K^+^ clearance from myelinated axons, was, in hindsight, predictable, given that myelin (typically) covers the majority of the surface area of axons, leaving little direct access for astrocytes to directly buffer ions (Figure 2). Indeed, several other ion channels/transporters including the solute transporter NKCC1 (Na^+^─K^+^─Cl^−^ cotransporter‐1) and the Na^+^─K^+^─ATPase are highly expressed in myelinating oligodendrocytes and, in principal, represent further factors that may be responsive to neuronal activity, whose regulation could in turn affect conduction and circuit function. In addition to the importance of K^+^ buffering for the regulation of function, it is clear that regulation of the concentrations and flux of other ions also has the potential to greatly affect the function of myelinated axons. In our own recent studies, we have found that the intracellular levels of Ca^2+^ in myelin sheaths can dynamically regulate both normal myelination and myelin pathology, which will in turn affect axonal function (Baraban et al., 2018). However, we have much to learn about the subcellular distributions of various Ca^2+^ channels and regulators of Ca^2+^ signalling along myelinated axons, both at the axonal and glial side. Furthermore, coordinated control of Na^+^ and Cl^−^ ions at the axon‐myelin interface are likely to influence myelinated axon function. For example, knock out of the chloride channel ClC‐2 gene generates white matter vacuolization similar to that seen for *K*_*IR*_4.1 channel global knock outs and increases in fluid between myelin and the axon (Blanz et al., 2007). In addition to needing to better understand how myelinating glia regulate ionic homeostasis along myelinated axons, how such ions may also be regulated by other glia in the broader network of the glial syncytium (Rash, 2010) largely remains to be investigated. In conclusion, myelinating oligodendrocytes likely serve numerous roles in ionic and metabolic homeostasis, but it is also possible that the mediators of these roles contribute to dynamic regulation of the function of the myelinated axon unit.

## 9 TOWARD THE FUTURE

We have provided an overview of the key components and domains of myelinated axons, focusing on their potential for extrinsic regulation by neuronal activity. As we alluded to throughout, it is very likely that the responses of the components of a myelinated axon to signals such as neuronal activity will neither occur in isolation, nor affect function in isolation. This theme is very much in its infancy and ongoing studies will need to consider myelinated axons as functional units, and also contend with the fact that different circuits are likely to exhibit great diversity in the nature of their myelinated axon composition and adaptability. For example, an attractive premise is that axons with partial myelination may retain a greater capacity for dynamic modulation, for example, in response to neuronal activity, over time, and that regulation along such axons might have the most prominent effect on conduction. As of now, however, our knowledge of how the formation or regulation of myelin along axons of the CNS occurs is limited, and how this affects conduction, rudimentary. Future studies will need to integrate highly accurate anatomical/morphological information of circuits at high resolution, ideally over time, with specific manipulations of the distinct components and constituents of myelinated axons, with detailed assessments of function, supported by computational modeling.

In addition to making the case that we need to consider myelinated axons as functional units, we highlight the fact that the physiology of the myelin sheath itself is vastly more complex than was previously thought, even until very recently. We are only now beginning to understand how the physiology of myelin, and connected networks of glia may influence neural circuit function. Although it is clear that dysregulation of myelin physiology has detrimental effects on the nervous system, it remains to be investigated to what extent myelin physiology might be dynamically regulated by ongoing neural circuit activity, and whether any such regulation is more likely to be employed to homeostatically maintain neural circuit function or to allow dynamic modulation of circuit function. Although future studies have much to elucidate, it is now clear that dynamic regulation of myelinated axons and myelin physiology play central roles in neural circuit function.

## CONFLICT OF INTEREST

The authors declare no conflict of interest.
